# Evaluation of anti-cancer effects of carnosine and melittin-loaded niosomes in MCF-7 and MDA-MB-231 breast cancer cells

**DOI:** 10.3389/fphar.2023.1258387

**Published:** 2023-09-21

**Authors:** Mohamed M. A. Hussein, Ahmed Abdelfattah-Hassan, Haitham Eldoumani, Walaa M. Essawi, Tariq G. Alsahli, Khalid Saad Alharbi, Sami I. Alzarea, Hassan Y. Al-Hejaili, Sara F. Gaafar

**Affiliations:** ^1^ Biochemistry Department, Faculty of Veterinary Medicine, Zagazig University, Zagazig, Egypt; ^2^ Department of Anatomy and Embryology, Faculty of Veterinary Medicine, Zagazig University, Zagazig, Egypt; ^3^ Biomedical Sciences Program, University of Science and Technology, Zewail City of Science and Technology, Giza, Egypt; ^4^ Department of Anatomy and Embryology, Faculty of Veterinary Medicine, Mansoura University, Mansoura, Egypt; ^5^ Department of Theriogenology, Faculty of Veterinary Medicine, Aswan University, Aswan, Egypt; ^6^ Department of Pharmacology, College of Pharmacy, Jouf University, Sakaka, Saudi Arabia; ^7^ Department of Pharmacology and Toxicology, Unaizah College of Pharmacy, Qassim University, Qassim, Saudi Arabia; ^8^ Pharmaceutical Care Department, King Salman Bin Abdulaziz Medical City, Ministry of Health, Medina, Saudi Arabia

**Keywords:** carnosine, melittin, niosome, breast cancer cells, cell cycle analysis, miRNA-183

## Abstract

**Background:** We investigated the anti-cancer effect of carnosine-loaded niosomes (Car-NIO) and melittin-loaded niosomes (Mel-NIO) with olaparib in breast cancer cell lines (MCF-7 and MDA-MB-231).

**Methods:** The thin film method was used for preparing the niosomes and characterized in terms of morphology, size, and polydispersity index (PDI). We further evaluated the impact of these peptides on breast cancer cells viability, RT-qPCR assays, malondialdehyde (MDA) activity, and cell cycle progression, to determine if these are linked to carnosine and melittin’s anti-proliferative properties.

**Results:** Car-NIO and Mel-NIO *in vitro* study inhibited cancer cell viability. They have also upregulated the expression of protein 53 (P53), BCL2-Associated X Protein (Bax), caspase-9, caspase-3, programmed cell death 4 (PDCD4), and Forkhead box O3 (FOXO3), while downregulated the expression of B-cell lymphoma 2 (Bcl2), poly (ADP-ribose) polymerase (PARP 1), and MicroRNA-183 (miRNA-183). The MCF-7 cells were arrested at the G2/M phase in Car-NIO, on the other hand, the MDA-MB-231 cells were arrested at the S phase. While the Mel-NIO and olaparib arrested the MCF-7 and MDA-MB-231 cells at the G0/1 phase.

**Conclusion:** Our study successfully declared that Mel-NIO had more anti-cancer effects than Car-NIO in both MCF-7 and MDA-MB-231 breast cancer cells.

## Graphical Abstract

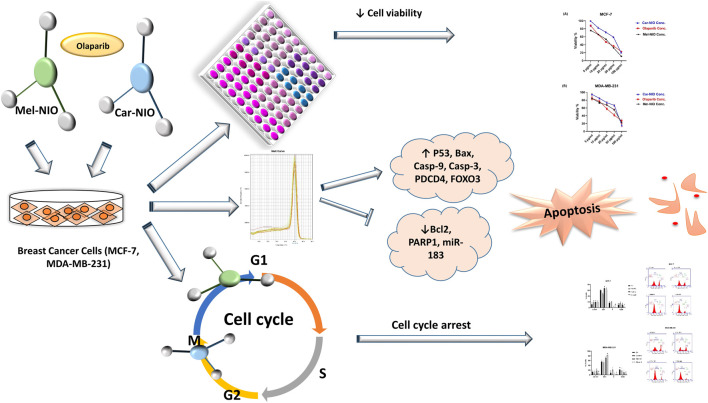

## 1 Introduction

A series of disorders known as cancer disrupt the genome, causes aberrant cell development and expands or encroaches on different body regions. Anticancer therapeutic peptide (ACP) medicines have distinct characteristics. Peptides have been thought to target and eliminate cancer cells. It has been established over time that ACPs have a variety of biological targets via which they act, including cell membranes (necrosis), mitochondrial membranes (apoptosis), and non-membrane action (Bakare et al., 2021). In actuality, the majority of ACPs work when they become internalized by cancer cells, although their ability to penetrate cancer cells is limited. To enable impactive therapy, it is crucial to increase the penetrability of ACPs. Numerous studies have already been conducted on various methods to improve ACPs’ ability to penetrate cancer cells (Ghaly et al., 2023).

Carnosine is a dipeptide molecule (β-alanyl-L-histidine) that occurs naturally and has anti-inflammatory, anti-oxidant, anti-glycosylation, and chelating impacts. Endogenous concentrations in humans can reach 20 mM, mostly found in the brain and skeletal muscle. The field of exercise physiology has long made use of this dietary supplement to boost performance (Hussein and Gaafar, 2022). However, there was limited *in vitro* proof that carnosine could specifically stop cancer cells from proliferating. The quick metabolism of carnosine by serum and kidney carnosinases is thought to be one of the factors limiting its impactiveness as a medication. These enzymes rapidly lower the serum level of carnosine, blocking its long-lasting impacts (Prakash et al., 2021).

Melittin (Mel), separated from bee venom, is an amphipathic peptide that is water soluble, contains 26 amino acids, and 6 favourable charges. It is the primary active ingredient in bees. Mel was used due to its wide bioactivity, which includes antibacterial, antiviral, anti-inflammatory, and anti-cancer activities. Mel has demonstrated great impactiveness in producing cell cycle arrest, apoptosis, necrosis, mitochondrial disruption, and stopping the angiogenesis of cancer cells (Tiwari et al., 2022). The injection of Mel, on the other hand, is linked to irritating reactions at the injection site, erythema, discomfort, and finally edema, as well as the necrosis of vascular endothelial cells. So, while the application of Mel therapy has been relatively constrained, strong prospects for continuous, regulated, and targeted medication administration to enhance therapeutic impacts and lessen side impacts are micro-or nanoparticle-based drug delivery systems. These methods allow therapeutic compounds to be encapsulated to prevent contact with their environment (Wang et al., 2022).

Nanotechnology is a cutting-edge scientific discipline that takes into account oddities at the nanoscale. Therapeutic alternatives that require precision administration have nanoparticles as a potential delivery mechanism. Niosomes are one-of-a-kind drug carriers synthesized by a combination of cholesterol and non-ionic surfactants. Niosomes can stand in for phospholipid vesicles since they provide the same stability for both hydrophobic and hydrophilic drugs while also being biocompatible, biodegradable, inexpensive to prepare, and so on. Noisome lessens the drug’s negative side impacts, namely, toxicity, and hemolysis (Bhardwaj et al., 2020).

The most significant tumor suppressor gene is protein 53 (P53). P53 is a key marker for cancer, and a higher level of P53 expression may improve the prognosis of the patient. Bcl2-Associated X Protein (Bax) is a member of the B-cell lymphoma 2 (Bcl2) protein family, which is crucial for either cell death or life (Kaloni et al., 2022). Overexpression of Bax causes cells to undergo apoptosis, indicating that Bax must be tightly regulated from transcription to post-translational processing for cells to survive. Bcl2 is an essential apoptosis-regulating protein. Protection from cell death caused by oncogenic and environmental stressors is provided by its extremely variable expression in several haematological cancers (Wolf et al., 2022). Important steps in initiating and concluding apoptosis are facilitated by members of the caspase family (Boice and Bouchier-Hayes, 2020).

Numerous cancer treatments enhance apoptosis by indirectly activating these caspases, killing the cancer cells. Apoptosis, regulation of cell proliferation, replication, and DNA damage repair are only a few of the many important biological processes in which poly (ADP-ribose) polymerase-1 (PARP-1) plays a role (Kumar et al., 2020). Multiple types of cancer cells have been found to express higher levels of PARP-1, and this upregulation has been linked to tumour progression (Boice and Bouchier-Hayes, 2020).

MicroRNAs (miRNAs) are a subclass of non-coding oligonucleotides that control gene expression in cancer cells. These modifications frequently result in alterations to cellular properties like proliferation, differentiation, and programmed cell death. In numerous cancer types, miRNA-183 has been demonstrated to have both tumor-suppressive and oncolytic functions (Hussein et al., 2021). miRNA-183 targets programmed cell death 4 (PDCD4) and Fork-head box O3 (FOXO3) to increase proliferation and invasion in human carcinoma (Cao et al., 2020; Rani et al., 2023).

Olaparib is a small molecule inhibitor of PARP that counteracts the effects of ionizing radiation and alkylating chemicals on DNA when taken orally. The FDA has approved the PARP inhibitor olaparib (Lynparza) to treat adult patients with deleterious or suspected deleterious gBRCAm, HER2-negative metastatic breast cancer who have been treated with chemotherapy in the neoadjuvant, adjuvant, or metastatic setting (Andreidesz et al., 2021).

This study aimed to reveal the chemoprotective impact of Car-NIO and Mel-NIO against *in vitro* cancer models by testing their ability to induce apoptosis on the MCF-7 and MDA-MB-231 breast cancer cells and investigating the mechanisms underlying this effect.

## 2 Materials and methods

### 2.1 Chemicals

Carnosine and melittin were purchased from Sigma Aldrich (St. Louis, MO, United States). Car-NIO and Mel-NIO were prepared at the Nanomaterials Research and Synthesis Unit, Animal Health Research Institute (ARC, Giza, Egypt). Olaparib was purchased from LKT Laboratories (St. Paul, MN, United States). The cell lines were purchased from the National Research Centre, Giza, Egypt. Other chemicals in the experiment were purchased from Sigma Aldrich (St. Louis, MO, United States).

### 2.2 Preparation of Car-NIO and Mel-NIO

#### 2.2.1 Preparation of Car-NIO

The thin film approach (Moulahoum et al., 2019) was used to prepare the niosomes, in a nutshell, 20 mg of cholesterol in 10 ML chloroform and 100 mg of Span 60 were mixed together. The solvent was removed using a rotary evaporator (Buchi R-3, Switzerland). Niosomes (Car-NIO) loaded with carnosine were created by hydrating the resultant thin film with a carnosine solution. To achieve a final concentration of 50 mg/mL, carnosine was dissolved in 10 mL of phosphate buffered saline (PBS) at Ph 7.4, 60°C. An ultrasonic bath (Sonics and Materials Inc., United States) was used to sonicate the aqueous solution and disperse the lipid layer at 60 HTz and room temperature for 15 min.

#### 2.2.2 Preparation of Mel-NIO

The thin-film approach was utilized in the creation of the Mel-NIO. A thin lipid film (120 rpm, 60°C, 1 h) was made by dissolving Span 60 (100 mg) and cholesterol (20 mg) in chloroform (10 mL) and then evaporating off the solvent with a rotary evaporator (Buchi R-3, Switzerland). The resultant thin film was then hydrated with a melittin solution to produce Mel-NIO. To achieve the desired final concentration, 10 mL of phosphate-buffered saline was added to separate solutions of melittin (50 mg/mL) at pH 7.4 at 60°C. The niosomal formulation was achieved by dispersing the lipid layer with the aqueous solution and sonicating it in an ultrasonic bath (Sonics and Materials Inc., United States) at 60 HTz and room temperature for 15 min (Moulahoum et al., 2019).

### 2.3 Characterization of Car-NIO and Mel-NIO

#### 2.3.1 Zeta potential, polydispersity index, and morphology

Dynamic light scattering (DLS) analysis was used to ascertain the niosomes size, polydispersity index (PDI) testing, and zeta potential (ZP) values (DLS, Malvern Zetasizer, Nano ZS model, Malvern Instruments Ltd., United Kingdom). Triplicate analyses were performed on each sample. The morphology of Car-NIO and Mel-NIO formulations was analyzed with the help of a digital micrograph and soft image viewer software using a transmission electron microscope (TEM) (JEOL JEM-2100; JEOL Ltd., Tokyo, Japan). One drop of drug-loaded niosomal dispersion was diluted 10 times with deionized water and then put on a carbon-coated copper grid for 1 min to help niosomes stick. Once the samples were dry at room temperature, they were examined with TEM without being stained (Darwesh et al., 2021).

#### 2.3.2 Entrapment efficiency (EE)

At 4,000 g for 30 min, the Car-NIO and Mel-NIO formulations were ultra-filtered over an Amicon Ultra-15 membrane (MWCO 30,000 Da). The drug-carrying niosomes awaited filtration in the upper chamber, while the free medicines were allowed to flow beyond the filter membrane. UV-visible spectroscopy (JASCO, V- 530, Tokyo, Japan) was used to determine the drug’s concentration at its highest absorbance wavelength (420 nm for Mel-NIO and 205 nm for Car-NIO). Each drug concentration was compared to a standard curve. The following equation was then used to determine the efficiency of encapsulation (Yadavar-Nikravesh et al., 2021):

$$\text{Efficiency of Enclosure }\left(\%\right)=\left[\left(A-B\right)/A\right]X\;100$$

where A is the initial concentration of the drug in the niosomal preparations and B is the amount of free drug in the filtrate.

#### 2.3.3 Drug release study

A dialysis bag (MWCO = 12 kDa) was used to compare 2 mL of free drugs, Car-NIO and Mel-NIO for drug release (*in vitro*). This bag was placed in a phosphate buffered saline (PBS) solution (50 mL, 1X, pH = 5.4) with slow stirring (50 rpm) at 37°C. At certain intervals, a portion of the PBS solution was removed and replaced with a new aliquot.

#### 2.3.4 Stability studies

To test the Car-NIO and Mel-NIO’s stability, we kept them in two distinct environments. Both 25 ± 1°C and 4 ± 1 C were used to keep the formulation for a month. After that, measurements were taken of its physical parameters (such as mean particle size (nm) and entrapment efficiency (EE)) at zero, fourteen, and 30 days.

### 2.4 Cell culture

Culture of MCF-7 and MDA-MB-231 cells were maintained at sub-confluence in 37°C, 5% CO2, and complete Roswell Park Memorial Institute-1640 medium added (catalog number: 11875093, Sigma-Aldrich, St. Louis, MO, United States), with 10% fetal bovine serum (catalog number:16000044, Sigma-Aldrich, St. Louis, MO, United States), penicillin/streptomycin (catalog number: 15140122, Sigma-Aldrich, St. Louis, MO, United States), and L-glutamine (catalog number: 25030081, Sigma-Aldrich, St. Louis, MO, United States).

### 2.5 *In vitro* cytotoxicity

The 3-(4,5-dimethylthiazol-2-yl)-2,5-diphenyl-2H-tetrazolium bromide (MTT) assay is a colorimetric method that uses the transformation of yellow MTT into purple formazan. The following procedures were carried out in a clean environment using MTT Assay Kit (Cell Proliferation) (catalog number: ab211091, Abcam, United Kingdom), with the use of a Laminar flow cabinet meeting biosafety standard II (Baker, SG403INT, Sanford, ME, United States), l0^4^ cells/well were subjected to varying doses of Car-NIO (5, 10, 25, 50, and 100 μM) (Tehrani et al., 2018) and Mel-NIO (5, 10, 25, 50, and 100 μM) (Yaacoub et al., 2021) and olaparib (5, 10, 25, 50, and 100 μM) (Ashmawy et al., 2017). After 48 h, we added 2.5 μg/mL of MTT to each well and incubated the plates at 37°C for another 4 h. After the formation of formazan crystals, 10% sodium dodecyl sulfate (200 μL/well) was used to dissolve the crystals. We measured the absorbance at 595 nm, and a positive control that causes 100% mortality under the same conditions was utilized. Using the following formula, the change in viability was given as a percentage was calculated:

$$\text{Cytotoxicity }\%=\left(\text{Extract reading}/\text{Negative control reading}\right)\;x100$$

Viability % = 100- Cytotoxicity%, The impact of each treatment is measured by its IC50 (the concentration at which 50% of the viability is inhibited).

### 2.6 Quantitative RT-PCR analysis

The cells’ total RNA was extracted with the help of the RNA Mini Kit from Ambion by Life Technologies by Thermo Scientific (catalog number: 12183018A). To ensure the integrity of the RNA samples, we used a NanoDrop^®^ ND-1000 Spectrophotometer from NanoDrop Technologies in Wilmington, Delaware, United States A High Capacity cDNA Reverse Transcription Kit (catalog number: 4374966) from Thermo Scientific was then used to amplify the cDNA. The Maxima SYBR Green qPCR Master Mix (2X) kit from Thermo Scientific, (catalog number: K0251), was used for real-time PCR amplification (Shehata et al., 2018). The housekeeping GADPH (for mRNA) or U6 (for miRNA) were used as a standard. The 2^−∆∆ct^ formula was used to calculate the levels of target gene expression (Livak and Schmittgen, 2001). Table 1 contains the sequences of the desired gene primers.

**TABLE 1:** Primers sequences used in this study.

Gene	Primers	Accession number
P_53_	F 5′- CCT​CAG​CAT​CTT​ATC​CGA​GTG​G-3′	NM_000546
	R 5′-TGG​ATG​GTG​GTA​CAG​TCA​GAG​C-3′	
Bax	F 5′- TCA​GGA​TGC​GTC​CAC​CAA​GAA​G -3′	NM_001291428
	R 5′- TGT​GTC​CAC​GGC​GGC​AAT​CAT​C -3′	
Bcl2	F 5′- ATC​GCC​CTG​TGG​ATG​ACT​GAG​T-3′	NM_000633
	R 5′- GCC​AGG​AGA​AAT​CAA​ACA​GAG​GC-3′	
Caspase-9	F 5′-GTT​TGA​GGA​CCT​TCG​ACC​AGC​T-3′	NM_001229
	R 5′-CAA​CGT​ACC​AGG​AGC​CAC​TCT​T-3′	
Caspase-3	F 5′- GGA​AGC​GAA​TCA​ATG​GAC​TCT​GG-3′	NM_004346
	R 5′-GCA​TCG​ACA​TCT​GTA​CCA​GAC​C-3′	
PARP 1	F 5′- CCA​AGC​CAG​TTC​AGG​ACC​TCA​T-3′	NM_001618
	R 5′- GGA​TCT​GCC​TTT​TGC​TCA​GCT​TC-3′	
PDCD4	F 5′- ACT​GTG​CCA​ACC​AGT​CCA​AAG​G-3′	NM_001199492
	R 5′- CCT​CCA​CAT​CAT​ACA​CCT​GTC​C-3′	
FOXO3	F 5′- TCT​ACG​AGT​GGA​TGG​TGC​GTT​G-3′	NM_001455
	R 5′- CTC​TTG​CCA​GTT​CCC​TCA​TTC​TG-3′	
GAPDH	F 5′- GTC​TCC​TCT​GAC​TTC​AAC​AGC​G -3′	NM_001256799
	R 5′- ACC​ACC​CTG​TTG​CTG​TAG​CCA​A -3′	
MiRNA-183	F 5′- ATG​GCA​CTG​GTA​GAA​TTC-3′	MIMAT0000261
	R 5′- GAA​CAT​GTC​TGC​GTA​TCT​C-3′	
U6	F 5′- CTC​GCT​TCG​GCA​GCA​CAT-3′	NR_004394.1
	R 5′- TTT​GCG​TGT​CAT​CCT​TGC​G-3′	

### 2.7 Detection of lipid peroxidation

For this assay, the cells (2 × 10^6^ cells/mL) were seeded onto 96-well culture plates to determine lipid peroxidation levels. Following a 48-h incubation with Car-NIO (48.83, 51.4 μM), Mel-NIO (21.23, 43.65 μM), and olaparib (23.31, 32.55 μM), cells were collected and washed twice with phosphate-buffered saline. The cells were harvested with a sonicator probe (VCX-130 W, Newtown, United States) on ice, and then disrupted by ultra-sonication for 5 s. Following the manufacturer’s guidelines for best results, the malondialdehyde (MDA) was detected in the cell extract using the MDA test kit (catalog number: ab287797, Abcam, United Kingdom). The microplate reader was used to determine the MDA concentration at a wavelength of 532 nm (Tecan, Mannedorf, Switzerland) (Gérard-Monnier et al., 1998).

### 2.8 Detection of the cell cycle stage

Overnight, the cells were cultured in 100-mm plates at a density of 2 × 10^6^ cells/mL per well using fresh culture media. Treatment with Car-NIO (48.83, 51.4 μM), Mel-NIO (21.23, 43.65 μM), and olaparib (23.31, 32.55 μM) for 48 h began the next day. After harvesting, the cells were fixed in 70% ethanol at 20°C for 12 h. After that, the cells were exposed to RNase (10 mg/mL) and propidium iodide (50 μg/mL) for 30 min. The cell cycle distribution was analyzed using flow cytometry, and the data were analyzed using FlowJo (TreeStar, Ashland, OR, United States) (Mei et al., 2019).

### 2.9. Statistical analysis

Graph Pad Prism 5 (Graph Pad Software, La Jolla, CA, United States) was used for the statistical analysis. Multiple group comparisons were made using one-way analysis of variance (ANOVA) and Tukey’s *post hoc* test for statistical significance. All figures are reported as the average standard error of at least three independent measurements. The level of significance chosen was less than 0.05.

## 3 Results

### 3.1 Car-NIO and Mel-NIO characterization

#### 3.1.1 Morphological characterization

**FIGURE 1:**
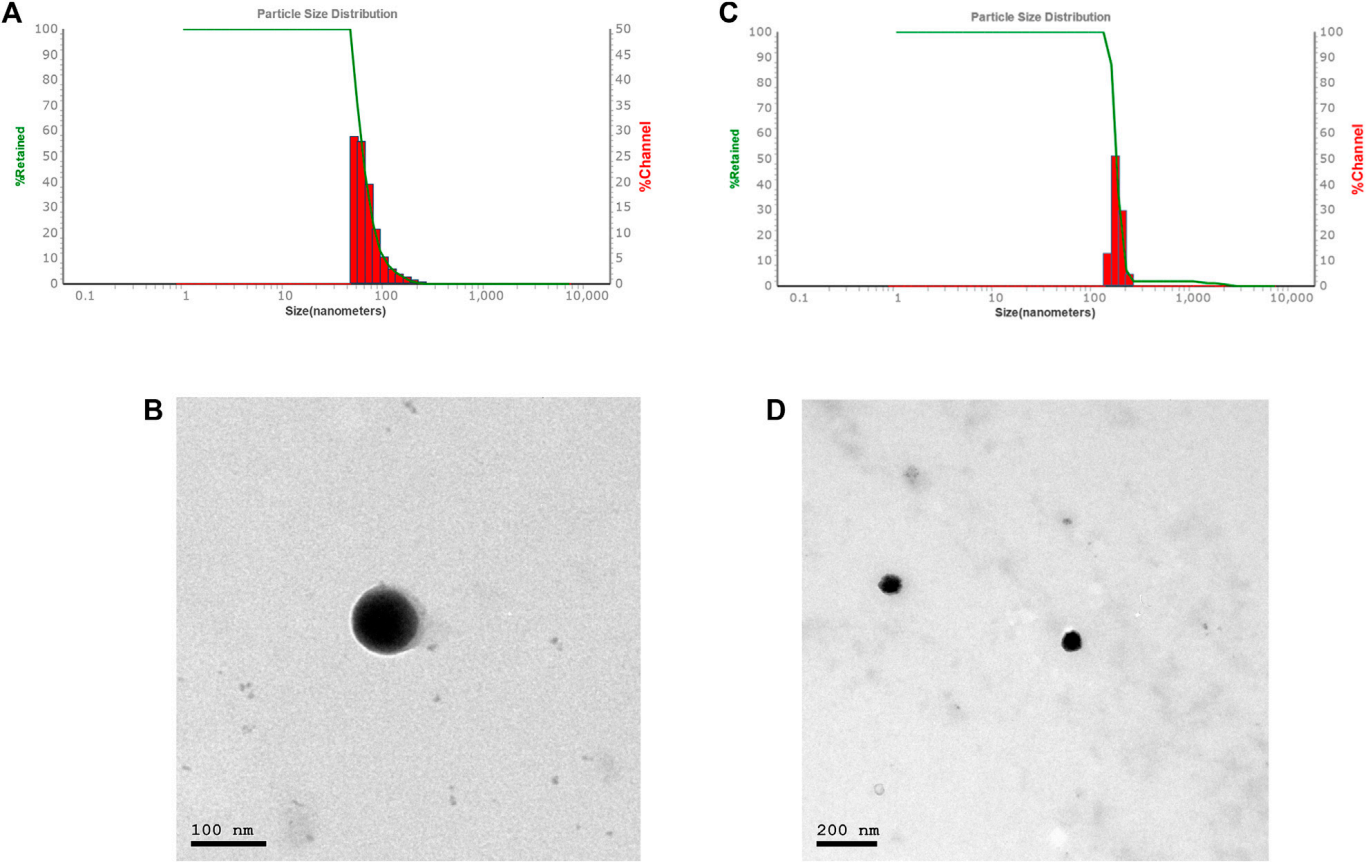
Characterization of Car-NIO and Mel-NIO. Dynamic light scattering size measurement of Car-NIO **(A)**. TEM image of the Car-NIO **(B)**. Mel-NIO dynamic light scattering size measurement **(C)**. TEM image of the Mel-NIO **(D)**.

The average particle size, PDI, and zeta potential were measured by dynamic scattering light analysis using the Malvern Zetasizer. In Car-NIO the average size was 58 ± 0.50 nm. The PDI value was 0.16441 ± 0.04, which is considered in the acceptable range. The ZP value of Car-NIO was −20 ± 0.3 mV (Figures 1A, B). While in Mel-NIO the average size was 163 ± 1.3 nm. The PDI value was 0.0424 ± 0.1, which is considered in the acceptable range. The ZP value of Mel-NIO was −86.6 ± 0.9 Mv (Figures 1C, D).

#### 3.1.2 Drug release studies of carnosine and melittin from niosomes

**FIGURE 2:**
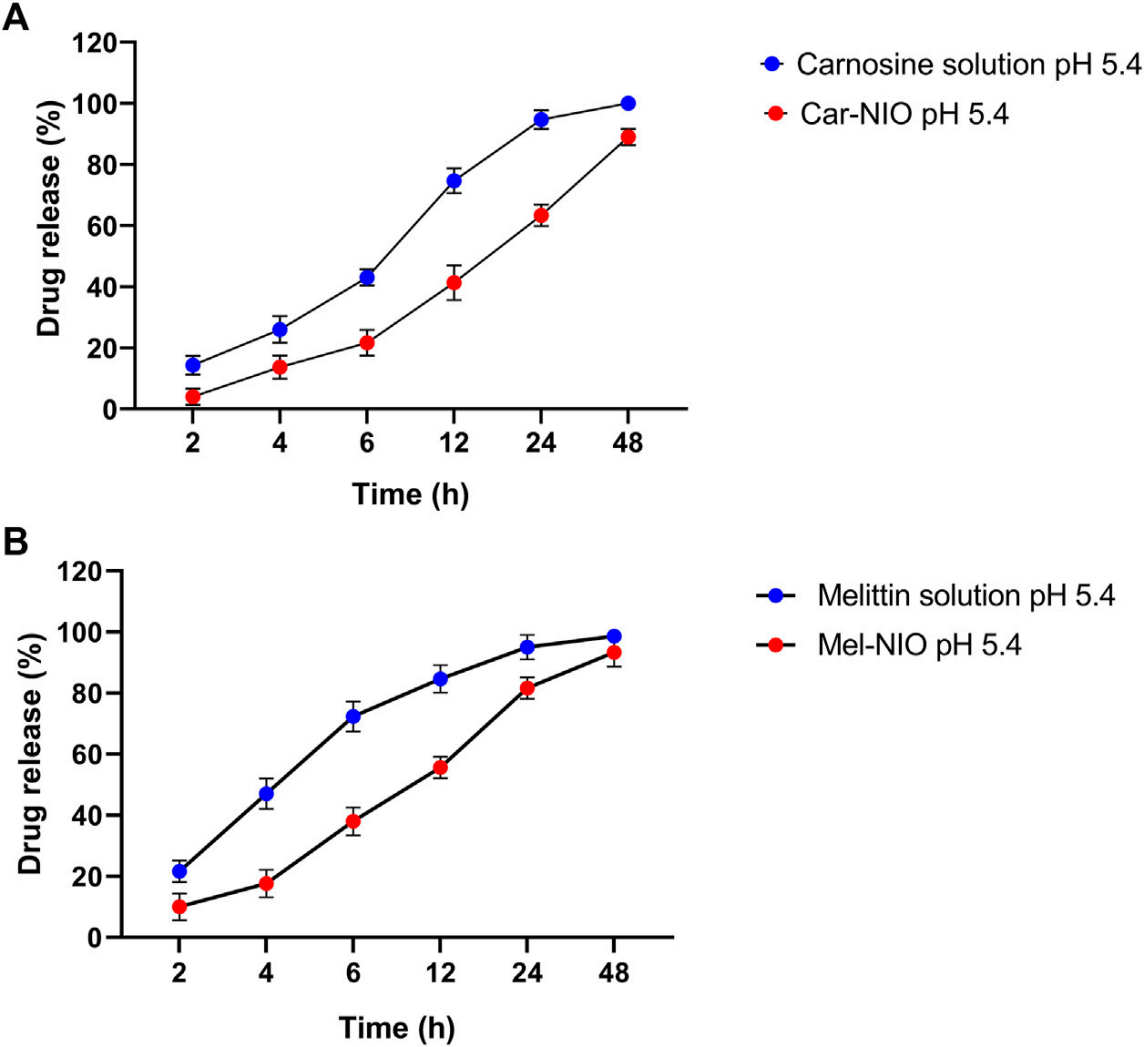
*In vitro* drug release profile of carnosine and Car-NIO **(A)**, melittin and Mel-NIO **(B)** from the dialysis bag in pH 5.4 at 37°C. Results are represented by mean ± SD (*n* = 3). *p < 0.05, **p < 0.001.

Each carnosine and melittin formulation’s drug release profile was studied for 48 h at 5.4 pH at 37°C to learn more about *in vitro* drug release. As can be observed in the “Release” plot (Figure 2), the free medications first saw a rapid increase in release (43%, 72.3% over the first 6 h); after 24 h, the rate of release slowed to a steady state. According to the monitoring of the Car-NIO and Mel-NIO release profile, after 6 h, 21.6%, and 38% of the drug had infiltrated cells at a pH of 5.4. According to research by Rinaldi et al. (Rinaldi et al., 2017), the expanding structure of niosomes in an acidic environment results in 89%, and 93.3% of the medications being released into the bloodstream after 48 h at pH 5.4, respectively. The acidic shift of niosomes has been linked to electrophilic addition processes. The release rate of the drugs inside the niosomes was carefully calculated. Tumour wards typically contain acidic conditions, which crushed the niosomes’ structure, accelerating the release rate and increasing the toxicity (Naderinezhad et al., 2017). More cytotoxicity is induced by an acidic environment because of changes in carnosine and melittin and an increase in osmotic pressure. Release statistics for melittin were calculated for 72 h at body temperature over 5.4 pH. Carnosine and melittin, as free drugs, were released at a rate, with an R2 value of 0.951 suggesting a release proportional to drug concentration.

#### 3.1.3 Physical stability study of Car-NIO and Mel-NIO

**FIGURE 3:**
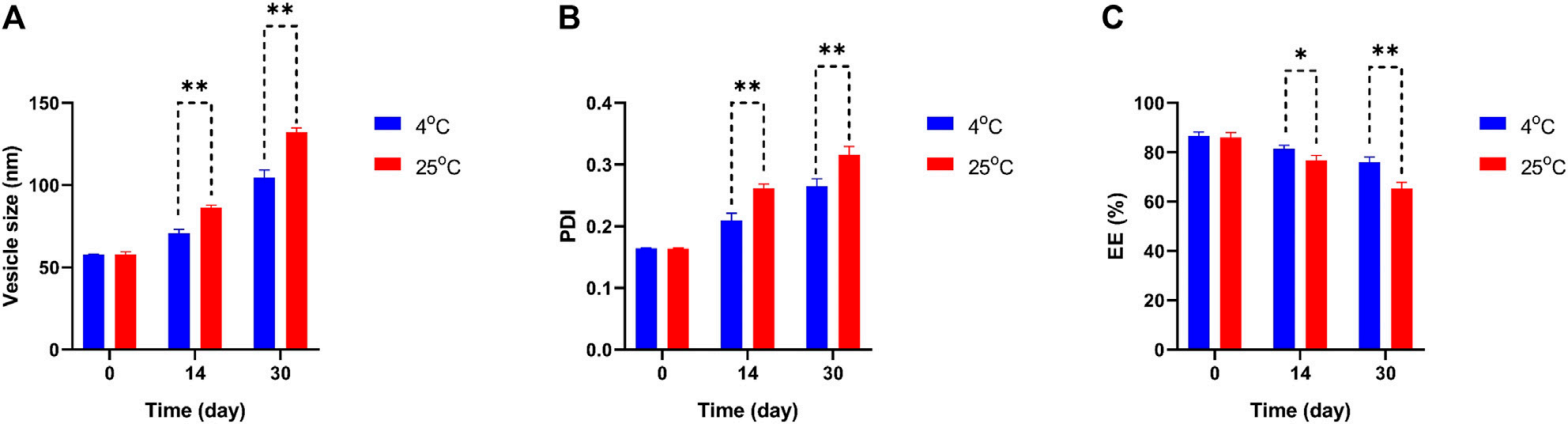
Comparing stability of optimum formulation at 4°C and 25°C of Car-NIO. Mean particle size **(A)**, PDI **(B)** and EE % **(C)** were studied as stability parameters. Results are represented by mean ± SD (*n* = 3). *p < 0.05, **p < 0.001.

To determine the physical stability and effectivity of Car-NIO and Mel-NIO, the vesicle size, PDI, and EE were measured at 4°C and 25°C, and on days 0, 14, and 30 following manufacture. Interestingly, the measurements showed that neither the particle size nor the PDI nor the EE percentage was impacted by the temperature and that the formulation recently generated possessed the least size of Car-NIO with a mean of 58 nm, the maximum PDI (0.316), and the EE (86.67%). The stability diagram (Figure 3) shows that the temperature had an effect on all the parameters from day 0 to day 30. Size growth, increased PDI, and diminished EE were all brought on by a rise in temperature. Increases in drug release as temperatures rise are responsible for the EE decrease (Nasseri, 2005). Growing the pores of the niosomes; could be beneficial on the particle size and PDI, increasing either of them and decreasing the EE to the lowest amount (76%), as the temperature can affect rigidity and elasticity. In contrast, at 25°C, the developed pores caused larger size, more PDI, and less EE (65.3%), suggesting that the niosomes are more stiff and elastomeric at lower temperatures.

**FIGURE 4:**
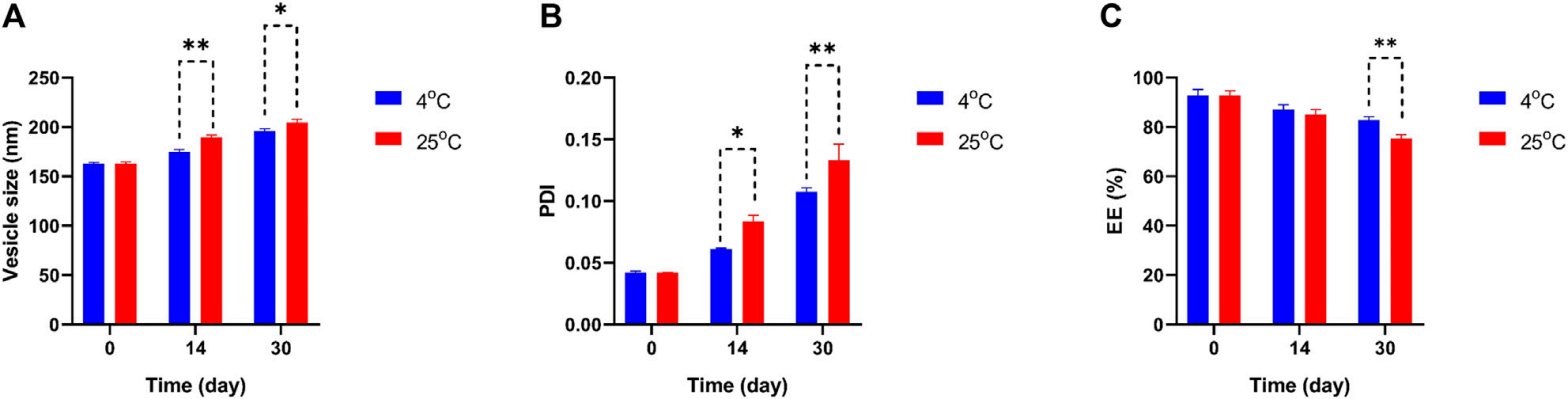
Comparing stability of optimum formulation at 4°C and 25°C of Mel-NIO. Mean particle size **(A)**, PDI **(B)** and EE % **(C)** were studied as stability parameters. Results are represented by mean ± SD (*n* = 3). *p < 0.05, **p < 0.001.

In Figure 4 the particle size, PDI, and EE percentage of Mel-NIO across a wide range of temperatures revealed some interesting trends. The Mel-NIO formulation had the smallest particles (mean size of 162.9 nm), highest PDI (0.133), and highest EE (92.6%). Temperature had an impact on all parameters from day 0 to day 30 as demonstrated by the stability diagram (Figure 4). A temperature rise led to an increase in size, PDI, and EE, and a decrease in EE. Temperature-induced increases in drug release explain the observed decline in EE. Increasing the niosomes’ pore size may improve the particle size and PDI, while decreasing the EE to its minimum value (82.6%) due to the temperature’s effect on the niosomes’ rigidity and elasticity. However, at 25 °C, the niosomes were found to be more rigid and elastomeric, with larger sizes, higher PDI, and lower EE (75.3%).

### 3.2 Cytotoxic impact on MCF-7 and MDA-MB-231 cells

**FIGURE 5:**
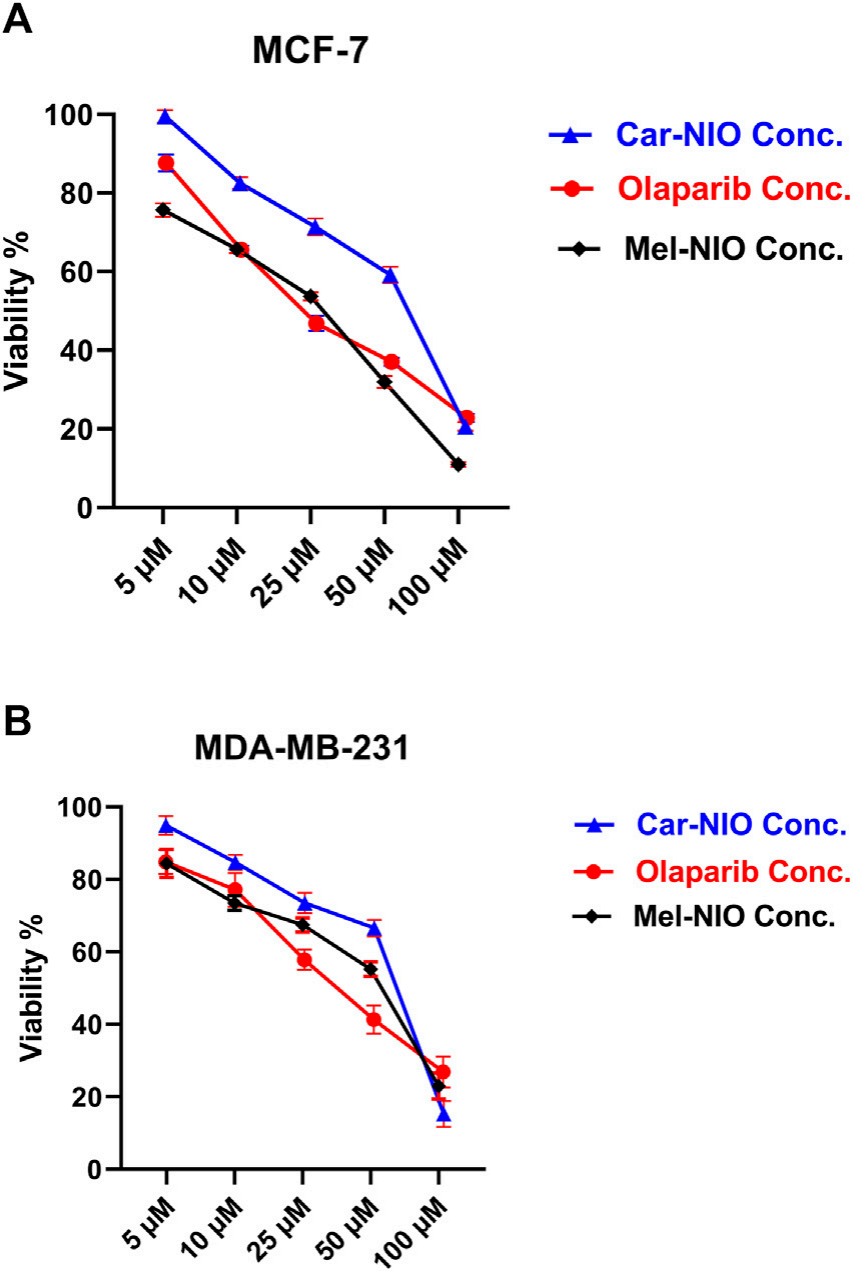
The impact of different concentrations of Car-NIO, Mel-NIO, and olaparib on the viability of MCF-7 **(A)**, and MDA-MB-231 **(B)** cells by using the MTT assay after 48 h of incubation. Data are shown as mean ± SEM, and *p* < 0.05.

Our results in Figure 5 referred to a dose-dependent basis, the viability% of MCF-7 and MDA-MB-231 cells were significantly reduced when the cells were incubated for 48 h with different concentrations of Car-NIO, Mel-NIO, and olaparib, respectively. Moreover, Car-NIO produced a cytotoxic effect on both cell types with an IC50 of 48.83, 51.4 μM, also in Mel-NIO with an IC50 of 21.23, 43.65 μM, and olaparib with IC50 of 23.31, 32.55 μM concentrations (Figures 5A, B).

### 3.3 Assessment of the mRNA expression levels of marker genes on MCF-7 and MDA-MB-231 cells

**FIGURE 6:**
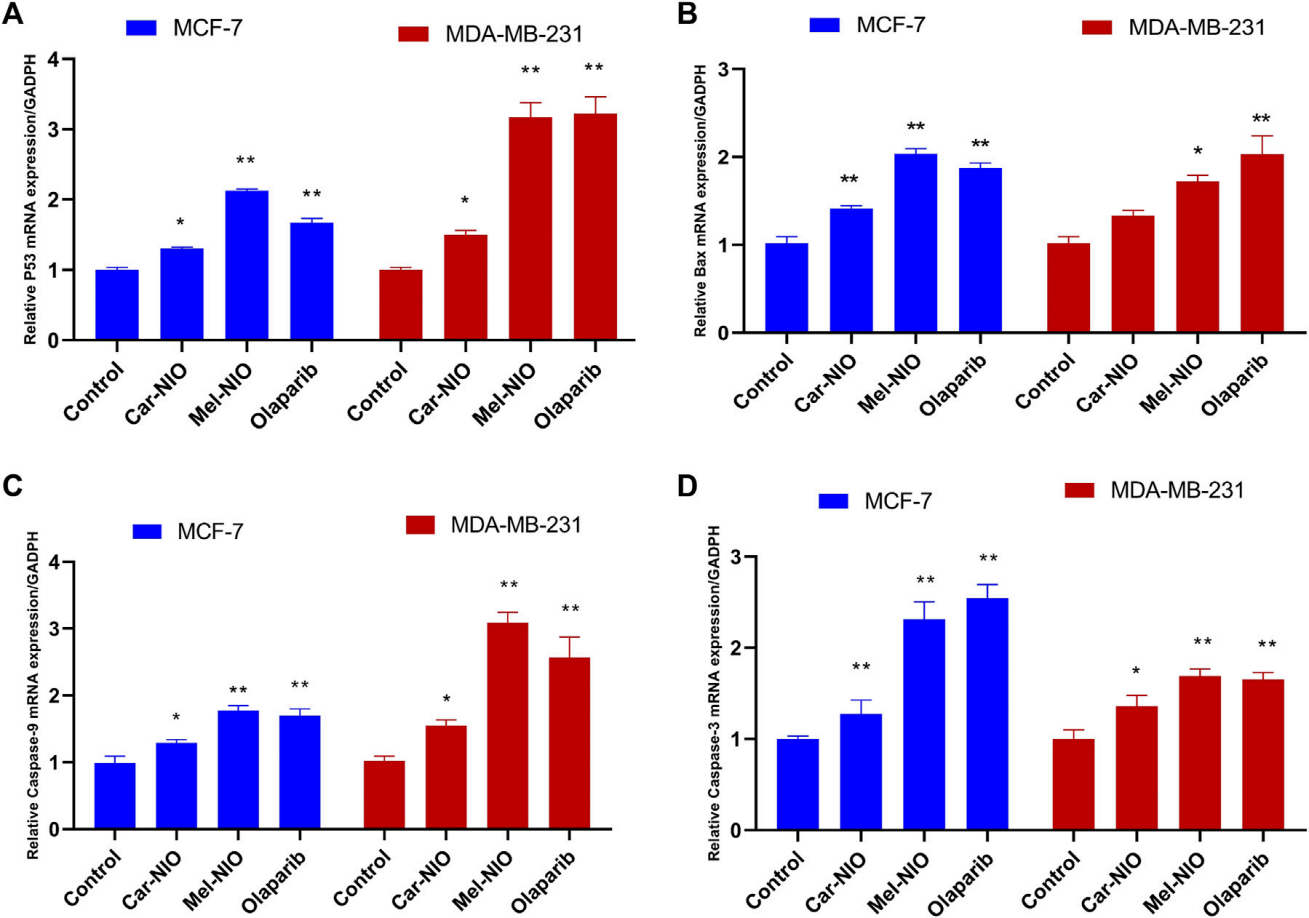
The impact of Car-NIO, Mel-NIO and olaparib on the genes expressions levels of P53 **(A)**, Bax **(B)**, caspase-9 **(C)**, and caspase-3 **(D)** (relative gene mRNA expressions/GADPH) in the MCF-7 and MDA-MB-231 cell lines. * Significant difference between control and treated group (*p* < 0.05), ** highly significant difference between control and treated group (*p* < 0.01).

Results showed a significant upregulation in the levels of mRNA expression of P53, Bax, caspase-9, and caspase-3 in MCF-7 and MDA-MB-231 cells treated with Car-NIO, Mel-NIO, and olaparib in comparison with untreated cancer cells. In MCF-7 cells treated with Mel-NIO and olaparib showed non-significant differences between each other in mRNA expression levels of caspase-9 and caspase-3, also non-significant differences in the expression level of caspase-3 between untreated cancer cells and Car-NIO treated cell were detected. While in MDA-MB-231 cells treated with Mel-NIO and olaparib showed non-significant differences between each other in the expression levels of P53 and caspase-3, on the other hand, the expression level of Bax showed non-significant differences between untreated cancer cells and Car-NIO treated cell (Figure 6).

**FIGURE 7:**
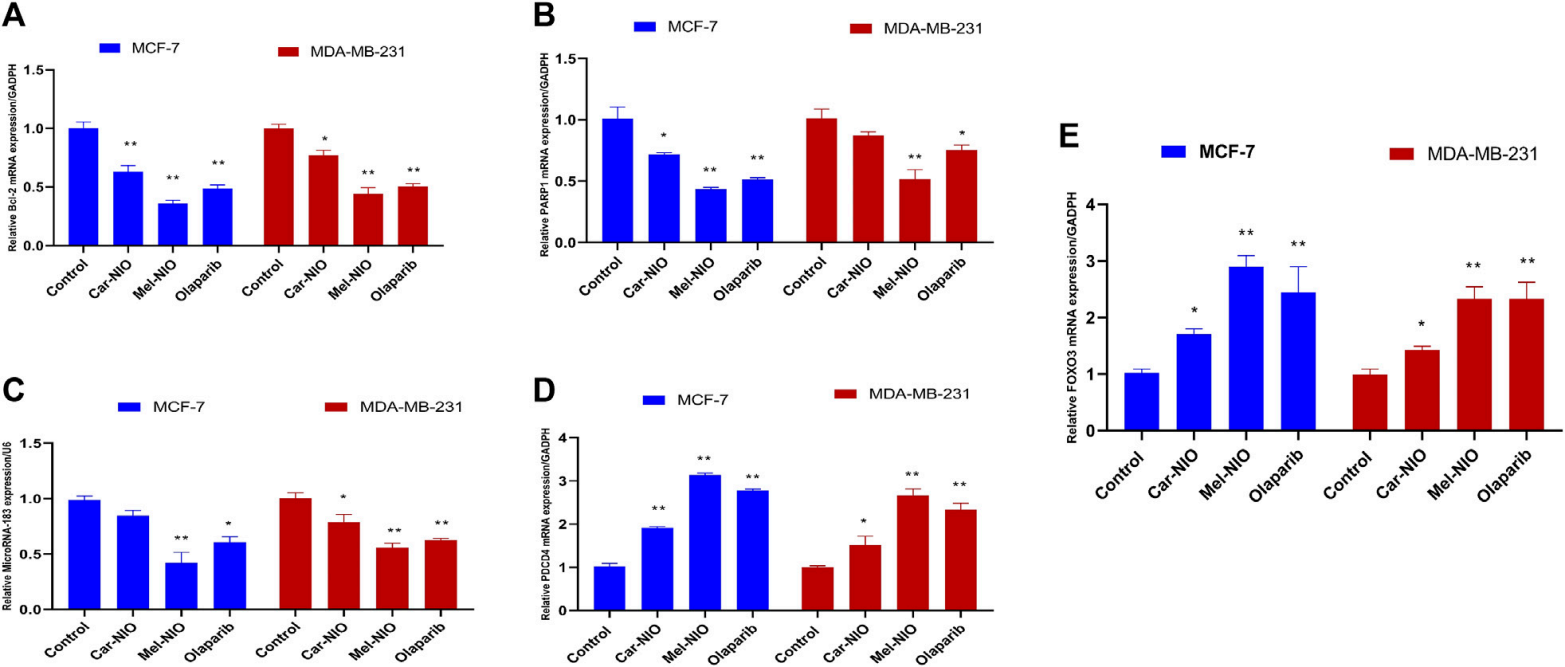
The impact of Car-NIO, Mel-NIO and olaparib on the genes expressions levels of Bcl2 **(A)**, PARP 1 **(B)**, MicroRNA-183 **(C)**, PDCD4 **(D)** and FOXO3 **(E)** (Relative gene mRNA expressions/GADPH) in the MCF-7 and MDA-MB-231 cell lines. * Significant difference between control and treated group (*p* < 0.05), ** highly significant difference between control and treated group (*p* < 0.01).

In contrast to our previous results, the expression levels of anti-apoptotic Bcl2, PARP 1, and miRNA-183 were significantly the highest transcriptional level in untreated cancer cells, while the cells treated with Car-NIO, Mel-NIO, and olaparib revealed significant downregulation in Bcl2, PARP 1, and miRNA-183 in MCF-7 and MDA-MB-231 cells in comparison with the untreated cancer cells. While, the expression levels of PDCD4 and FOXO3 were significantly upregulated in both cell types treated with Car-NIO, Mel-NIO, and olaparib in comparison with untreated cancer cells. Also, the FOXO3 expression level showed non-significant differences between Mel-NIO and olaparib-treated cells in both cell types. The expression levels of Bcl2, PDCD4, and miRNA-183 showed non-significant differences between Mel-NIO and olaparib-treated cells in MDA-MB-231 cells (Figure 7).

### 3.4 Assessment of lipid peroxidation marker (MDA)

**FIGURE 8:**
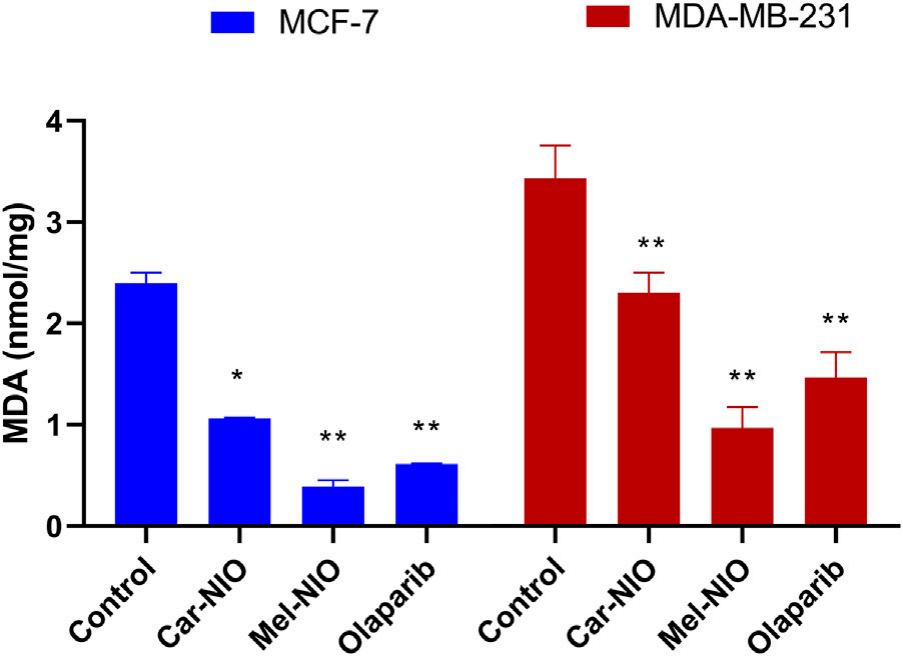
The impact of Car-NIO, Mel-NIO, and olaparib on the level of MDA (nmol/mg) in the MCF-7 and MDA-MB-231 cell lines. * Significant difference between control and treated group (*p* < 0.05), ** highly significant difference between control and treated group (*p* < 0.01).

The MDA levels were significantly higher in the untreated cancer cells, while the cells treated with Car-NIO, Mel-NIO, and olaparib showed significant downregulation in MCF-7 and MDA-MB-231 cells in comparison with the untreated cancer cells. On the other hand, in MDA-MB-231 cells the Mel-NIO, and olaparib-treated cells demonstrated non-significant differences in MDA levels (Figure 8).

### 3.5 Analysis of the cell cycle

**FIGURE 9:**
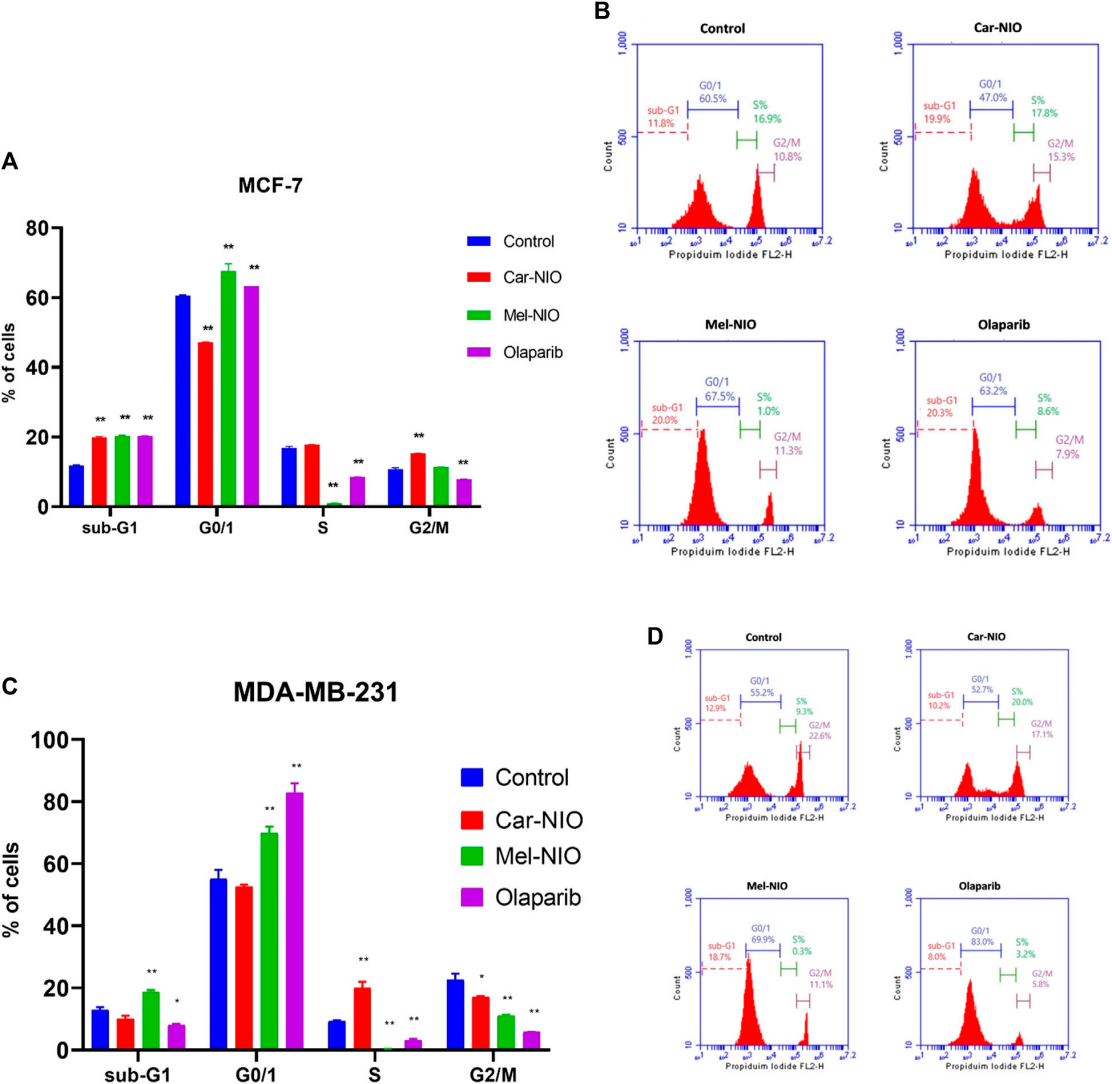
The impact of Car-NIO, Mel-NIO, and olaparib on cell cycle progression of breast cancer MCF-7 **(A, B)**, and MDA-MB-231 **(C, D)** cell lines. * Significant difference between control and treated group (*p* < 0.05), ** highly significant difference between control and treated group (*p* < 0.01).

We hypothesized that Car-NIO and Mel-NIO induced apoptosis involved cell cycle interruption, and thus evaluated the cell cycle distribution upon Car-NIO and Mel-NIO treatment. Breast cancer MCF-7 cells treated with Car-NIO for 48 h revealed a significant (*p* < 0.05) increase in the number of cells arrested at the G2/M (15.3%) phase, and the proportion of cells in the G0/1 phase (47.0%) was decreased in comparison with untreated cancer cells as well as cells treated with Mel-NIO showed a significant increase in the number of cells arrested at the G0/1 (67.5%) phase, and the proportion of cells in the S phase (1.0%) was decreased in comparison with untreated cancer cells. In the cells treated with olaparib, our data showed a significant increase in the number of cells arrested at G0/1 (63.2%), and the proportion of cells in the S phase (8.6%) was decreased in comparison with untreated cancer cells (Figures 9A, B).

Breast cancer MDA-MB-231 cells treated with Car-NIO for 48 h showed a significant increase in the number of arrested cells at S (20.0%) phase, and the proportion of cells in G2/M phase (17.8%) was decreased in comparison with untreated cancer cells as well as the cells treated with Mel-NIO showed a significant increase in the number of cells arrested at the Sub-G1 population (18.7%), and G0/1 (69.9%) phases and the proportion of cells in the S phase (0.3%) was decreased in comparison with untreated cancer cells. In the cells treated with olaparib, there was a significant increase in the number of cells arrested at the G0/1 (83.0%), and the proportion of cells in the S phase (3.2%) was decreased in comparison with untreated cancer cells. These findings in MCF-7 and MDA-MB-231 cells point to a block in the cell cycle at G1, and a decrease in the percentage of cells in S phase suggests fewer cells are entering the DNA-synthesis phase (Figures 9C, D).

## 4 Discussion

Finding a powerful anticancer medicine without harmful side impacts is still very much in demand. Bioactive peptides are abundant in a variety of food sources and are produced from food proteins by fermentation, enzymatic, chemical hydrolysis, or gastrointestinal digestive processes. In recent years, there has been a significant increase in the number of articles highlighting their potential benefits on blood pressure and lipid metabolism, in addition to their anticancer, immunomodulatory, antibacterial, analgesic, anti-oxidant, and anti-inflammatory actions (Komarov et al., 2022).

Even if the advantages of bioactive peptides are well known, there is still an opportunity for advancement because science is an area that is constantly evolving. Some chemicals degrade before they reach their targets or are unable to reach particular organs. Engineered carriers are an appealing strategy, especially when trying to treat cancer cells (Liu and Zhang, 2022). As an alternative to liposomes, niosomes are biodegradable, non-toxic, more stable, and less expensive vesicles made from non-ionic surfactants. Having a structure comparable to that of a liposome, they can stand in for liposomes as an alternate vesicular system. Niosomes have a propensity for loading many medications. One of their main advantages is that they can encapsulate chemicals of different physicochemical properties and modify the impacts these compounds have on the body (Alqosaibi, 2022).

Carnosine has long been recommended for usage as an antioxidant and antiglycating agent. There have also been indications of carnosine’s anti-proliferative activities, which may help prevent the emergence of a variety of malignancies. However, because serum and tissue carnosinase enzymes are present in the body, carnosine turns over quickly, necessitating repeated daily dosages for usage as a dietary supplement (Turner et al., 2021).

According to several reports, carnosine, for instance, slowed the formation of tumors in the fibroblast cells and hepatocellular carcinoma cells implanted in BALB/c nude mice model. When carnosine was administered concurrently with the subcutaneous injection of cancer cells into the dorsal skin of female nude mice, it has been shown to have a significant ability to inhibit the proliferation of malignant cells *in vivo*. Carnosine has been touted as having the advantage of slowing tumor growth, nevertheless (Horii et al., 2012).

Our findings demonstrated that Car-NIO and Mel-NIO had a protective impact against *in vitro* cancer trials, in which our treatments demonstrated a significant cytotoxic impact in MCF-7 and MDA-MB-231 breast cancer cells and upregulated the mRNA expression levels of P53, Bax, caspase-9, caspase-3, PDCD4, FOXO3 and downregulated the expression levels of Bcl2, PARP 1 and miRNA-183 in comparison with untreated cancer cells. This is the first publication we’re aware of that compares the chemo-protective effects of Car-NIO with Mel-NIO in many breast cancer cell lines together.

The carnosine derivative was combined with various concentrations of phosphatidyl-choline and cholesterol to create liposomal/carnosine conjugates, which were subsequently used to create liposomes. The resulting immunoliposomes, which had a size of about 100 nm, were subsequently examined *in vitro* in different cancer models. The liposomal/carnosine had higher IC50 values than the reference drug 5-Fluororuracil for all cell lines. Carnosine encapsulated in these liposomes is better protected from the impacts of carnosinase (Zoppo et al., 2016).

Gaafar et al. (Gaafar et al., 2021), coupled liquid crystalline nanoparticles (NPs) that are PEGylated with L-carnosine (P-LCNPs), and proved that P-Liquisomes were successful in maximizing the therapeutic impact of carnosine without changing its activity and may be employed as a delivery strategy for additional encouraging hydrophilic anticancer drugs. By *in vitro* analysis, spherical, light-colored vesicles encased in the liquid crystals were discovered, demonstrating the P-LCNPs were nanosized (149.3 nm) with high ZP. After 24 h of incubation, *in vitro* cytotoxicity testing showed that P-Liquisomes had a superior cytotoxic impact. Also, compared to carnosine solution, P-Liquisomes had a stronger chemopreventive impact, as shown by a decrease in tumor growth, an increase in the ratio of tumor growth inhibition %, vascular endothelial growth factor levels, cyclin D1 levels, and caspase-3 levels, and a decrease in tumor size.

Reactive oxygen species (ROS) are a form of oxidative stress that has been linked to carcinogenesis. It plays a role in DNA damage, cell proliferation, adhesion, survival, and apoptosis, among other physiological and pathological processes. MDA is commonly utilized as a biomarker of oxidative stress during severe health problems like cancer, etc., due to its status as a lipid peroxidation marker caused by ROS that is both extremely cytotoxic and carcinogenic. MDA, or lipid peroxidation, is elevated in several types of tumours. There is evidence that it encourages cellular senescence and death. During times of oxidative stress, MDA is a functional intermediate that contributes to cellular growth. Since MDA interactions with amino groups are a naturally occurring metabolic process, it has been hypothesized that these changes may be responsible for proliferation. Excessive ROS generation was linked to DNA damage, heritable mutations, mitochondrial dysfunction, and cell death. Moreover, a differential redox control in proliferation and viability between normal and malignant cells suggests that tumour cells are more vulnerable to oxidative stress than healthy cells. One potential therapeutic strategy involves exploiting the redox fragility of cancer cells. Increased metabolic stress combined with strong proliferative capacity causes these cells to lose their ability to effectively regulate high levels of induced cellular oxidative stress, leading to elevated levels of reactive oxygen species-associated DNA damage and ultimately compromising cell viability. The cells treated with Car-NIO and Mel-NIO especially Mel-NIO showed non-significant differences in some parameters in comparison with cells treated with olaparib. The cell cycle progression results suggested that the cells treated with Car-NIO and Mel-NIO arrested a high proportion of cells in the G1 phase as well as the reduction of cells in the S phase indicating the reduction of the number of cells entering the phase of DNA synthesis in both cell lines.

Our results are in line with preceding reports suggesting that carnosine prohibits the G1-S stage progress in both HeLa (adenocarcinoma) and SiHa (squamous cell carcinoma) cells, causing G1 arrest. Based on all the evidence, it looks like carnosine stopped the growth of human cervical gland carcinoma cells (Bao et al., 2018).

In murine bone marrow cells, carnosine attenuated cyclophosphamide-induced G2/M arrest, which was associated with decreased phosphorylated-checkpoint kinase 1/checkpoint kinase 1 and phosphorylated-P53/P53 ratios as well as decreased protein 21 expressions. Carnosine therapy also prevented cell death brought on by cyclophosphamide (Deng et al., 2018).

Melittin is the leading active ingredient in bee venom and has a wide range of biological functions. However, because of substantial side impacts, the researchers created melittin loaded on nanocarriers to reduce toxicity and investigate the inhibitory actions on liver cancer along with biological safety. It was suggested that melittin nano-liposomes are a bright new drug for treating hepatocellular carcinoma (Mao et al., 2017).

In the past, Melittin has been encapsulated using a wide variety of nanocarrier delivery technologies, such as liposomes, polymeric or solid lipid nanoparticles, lipid or albumin nanocapsules, micelles, etc. Niosome-based drug delivery systems have been developed as an alternative to liposome-based systems because of the instability of liposome structures, which can result in drug leakage. Despite their structural differences, niosomes (also known as non-ionic surfactant vesicles) share many of the same activities and physicochemical properties as liposomes. Since subsequent studies confirmed that the melittin and carnosine-loaded nanocarrier demonstrated greater efficacy, bioavailability, fewer side effects, and a more potent anticancer effect than the free form, we decided against using the free form as a comparison and instead used two different kinds of peptides and a new standard drug in our experiment (Tian et al., 2011; Huang et al., 2013; Mao et al., 2017).

Tian et al. (2011) examined the anti-cancer impact and vascular stimulation of melittin liposomes and a poloxame with melittin solution. Results showed that melittin liposomes with a poloxamer-modified surface had greater bioavailability, more effective anticancer action, and fewer adverse effects than melittin solution. The melittin may accumulate in the tumor location as a result of the prolonged circulation period and enhanced permeability and retention action of liposomes, increasing its anti-tumor efficacy.

To mitigate the toxicity, allergic responses, and pain caused by free melittin, Mao et al. (2017) developed melittin nano-liposomes by encapsulating melittin with poloxamer. They looked into the biological safety and the inhibitory effects on liver cancer. They found that melittin nano-liposomes significantly reduced tumour formation following orthotopic and subcutaneous hepatocellular carcinoma (HCC) transplantation *in vivo* and suppressed HCC cell survival *in vitro*, in comparison to free melittin. An important finding was that it reduced inflammation and allergy symptoms in mice compared to melittin. When compared to free melittin, melittin nano-liposomes have higher anti-tumor efficacy and enhanced biological safety, making them a promising candidate for use in HCC therapy.

Huang et al. (2013), explained that the melittin-based lipid nanoparticle had a prospective clinical use in solid tumor therapies through intravenous injection due to its good characteristics. This melittin-based lipid nanoparticle hides the positive charge of melittin within the phospholipid monolayer, generating a neutral nanoparticle with lower cytotoxicity and a wider safe dose range. Finally, melanoma-carrying mice were intravenously injected with melittin-NPs. The melittin-NPs inhibited the proliferation of the melanoma cells in comparison with a control group. In addition, the core-shell spherical morphology of melittin-NPs allows for the synergistic loading of chemical agents, their perfect bio-compatibility and monodispersity give them superior utility as synergistic therapeutics, and their ultrasmall size makes it possible for them to efficiently penetrate solid tumours.

The melittin-encoded lipid-coated polymeric nanoparticle complex significantly inhibited tumor development without causing hemolysis or tissue damage. The outcome showed that the core-shell-structured melittin nanoparticle that was made may be able to solve the main problems melittin faces in clinical applications and has a lot of potential to be used to treat cancer (Ye et al., 2021).

Asfour et al. (2022), suggested that in diabetic rats, the nanoconjugate of gabapentin and melittin demonstrated remarkable wound healing properties. The size of the complex was 156.9 nm. The results of the *in vivo* investigation declared that mice treated with gabapentin-melittin had faster wound healing. The complex formula demonstrated antioxidant properties by increasing the levels of glutathione peroxidase and superoxide dismutase while inhibiting the accumulation of MDA.

Consistent with previous research, another study found that melittin significantly upregulates the caspase-2 and Bax, and depresses Bcl2 protein expression in comparison with untreated cancer cell line tests and *in vivo* model of lung cancer, by activating caspase-2 by suppressing miRNA-183 expression. When patients with lung cancer were given an injection under the skin, the size and weight of their tumors were much smaller in the melittin group than in the vehicle group (Gao et al., 2018).

The oral PARP inhibitor olaparib is therapeutically impactive in patients with recurrent ovarian cancer and a breast cancer gene (BRCA) mutation. People who have human epidermal growth factor receptor 2-negative, metastatic breast cancer and a hereditary BRCA mutation benefited greatly from switching to olaparib monotherapy rather than the standard treatment, with olaparib monotherapy extending median progression-free survival by 2.8 months and reducing the risk of death (Tutt et al., 2021).

Moskwa et al. (2011), suggested that the miRNA-182 overexpression was sensitive to the PARP inhibitor *in vitro* and *in vivo*. A clinical-grade PARP1 inhibitor affected the development of cancers in animal models that express miRNA-182. Together, these results show that the downregulation of BRCA1 by miRNA-182 stops DNA repair and may affect how breast cancer is treated.

Ashmawy et al. (2017), proved that miRNAs-181a/b were suppressed by their inhibitors followed by treatment with olaparib. The MDA-MB-231 cells revealed a large increase in cell survival, cell proliferation, and ATM protein as well as a significant decrease in Bcl2 activity. Their results demonstrated that miRNA-181a and miRNA-181b are essential for determining the olaparib sensitivity of triple-negative breast cancer cells. Additionally, miRNAs181a/b may be employed as a potential predictive biomarker for olaparib response.

According to research, miRNA-183 can restrict PDCD4 expression and hence prevent the apoptosis of transforming growth factor beta 1-induced human hepatocellular carcinoma cells, also miRNA-183 is suggested to restrict FOXO expression in lung cancer (Li et al., 2010; Zhang et al., 2015). Based on another study, it was concluded that miRNA-183 played a crucial role in the advancement of cancer cells by promoting oesophageal squamous cell carcinoma cell proliferation and invasion via binding to the PDCD4 mRNA (Ren et al., 2015) and this was in parallel with our results.

The carnosine and melittin nanoformulations for the treatment of breast cancer utilizing a stimulus-responsive system are promising and provide fresh information on the chemotherapeutic usage of carnosine and melittin. This study also highlights the potential of natural medicines with specific nanoformulation to replace costly and potentially harmful conventional cancer chemotherapeutics. Last but not least, even though our formulation’s potential as a diagnostic tool needs further work, it is still a fascinating future project with considerable potential therapeutic consequences.

## 5 Conclusion

In this study, we observed that Car-NIO and Mel-NIO significantly inhibited the proliferation of MCF-7 and MDA-MB-231 breast cancer cell lines, but the Mel-NIO showed significantly greater anti-cancer activity on these breast cancer cells compared to Car-NIO. Our data revealed additional impacts of Car-NIO and Mel-NIO by upregulating the levels of P53, Bax, caspase-9, caspase-3, PDCD4, FOXO3 and downregulating the expression of Bcl2, PARP 1, and miRNA-183, which also decreased MDA levels, Car-NIO inhibited the cells at the G2/M phase transition in MCF-7 cells and S phase at MDA-MB-231 cells, while Mel-NIO and olaparib inhibited both cells at the G0/1 phase transition and occur inhibition of cells at S phase. All these implications indicate the anti-proliferative properties of Car-NIO and Mel-NIO, which could be useful on MCF-7 and MDA-MB-231 breast cancer cells.

## Data Availability

The original contributions presented in the study are included in the article/Supplementary material, further inquiries can be directed to the corresponding author.

## References

[B1] Alqosaibi A. I. (2022). Nanocarriers for anticancer drugs: Challenges and perspectives. Saudi J. Biol. Sci. 29, 103298. 10.1016/j.sjbs.2022.103298 35645591 PMC9130109

[B2] Andreidesz K. Koszegi B. Kovacs D. Bagone Vantus V. Gallyas F. Kovacs K. (2021). Effect of oxaliplatin, olaparib and LY294002 in combination on triple-negative breast cancer cells. Int. J. Mol. Sci. 22, 2056. 10.3390/ijms22042056 33669671 PMC7921931

[B3] Asfour H. Z. Alhakamy N. A. Ahmed O. A. Fahmy U. A. El-Moselhy M. A. Rizg W. Y. (2022). Enhanced healing efficacy of an optimized gabapentin-melittin nanoconjugate gel-loaded formulation in excised wounds of diabetic rats. Drug Deliv. 29, 1892–1902. 10.1080/10717544.2022.2086943 35748413 PMC9246110

[B4] Ashmawy A. M. Sheta M. A. Zahran F. Abdel Wahab A. H. (2017). MiRNAs-181a/b as Predictive biomarkers for olaparib sensitivity in triple-negative breast cancer cells. BLJ 13, 221–229. 10.21608/blj.2017.47612

[B5] Bakare O. O. Gokul A. Wu R. Niekerk L. A. Klein A. Keyster M. (2021). Biomedical relevance of novel anticancer peptides in the sensitive treatment of cancer. Biomolecules 11, 1120. 10.3390/biom11081120 34439786 PMC8394746

[B6] Bao Y. Ding S. Cheng J. Liu Y. Wang B. Xu H. (2018). Carnosine inhibits the proliferation of human cervical gland carcinoma cells through inhibiting both mitochondrial bioenergetics and glycolysis pathways and retarding cell cycle progression. Integr. Cancer Ther. 17, 80–91. 10.1177/1534735416684551 28008780 PMC5950946

[B7] Bhardwaj P. Tripathi P. Gupta R. Niosomes P. S. (2020). Niosomes: A review on niosomal research in the last decade. J. Drug Deliv. Sci. Technol. 56, 101581. 10.1016/j.jddst.2020.101581

[B8] Boice A. Bouchier-Hayes L. (2020). Targeting apoptotic caspases in cancer. Biochim. Biophys. Acta - Mol. Cell. Res. 1867, 118688. 10.1016/j.bbamcr.2020.118688 32087180 PMC7155770

[B9] Cao D. Di M. Liang J. Shi S. Tan Q. Wang Z. (2020). MicroRNA-183 in cancer progression. J. Cancer 11, 1315–1324. 10.7150/jca.39044 32047538 PMC6995398

[B10] Darwesh A. Y. El-Dahhan M. S. Meshali M. M. (2021). A new dual function orodissolvable/dispersible meclizine HCL tablet to challenge patient inconvenience: *In vitro* evaluation and *in vivo* assessment in human volunteers. Drug deli. Transl. Res. 11, 2209–2223. 10.1007/s13346-020-00889-z 33443718

[B11] Deng J. Zhong Y. F. Wu Y. P. Luo Z. Sun Y. M. Wang G. E. (2018). Carnosine attenuates cyclophosphamide-induced bone marrow suppression by reducing oxidative DNA damage. Redox Biol. 14, 1–6. 10.1016/j.redox.2017.08.003 28826042 PMC5565745

[B12] Gaafar P. M. El-Salamouni N. S. Farid R. M. Hazzah H. A. Helmy M. W. Abdallah O. Y. (2021). Pegylated liquisomes: A novel combined passive targeting nanoplatform of L-carnosine for breast cancer. Int. J. Pharm. 602, 120666. 10.1016/j.ijpharm.2021.120666 33933646

[B13] Gao D. Zhang J. Bai L. Li F. Dong Y. Li Q. (2018). Melittin induces NSCLC apoptosis via inhibition of miR-183. Onco Targets Ther. 1, 4511–4523. 10.2147/OTT.S169806 PMC6078185 30122943

[B14] Gérard-Monnier D. Erdelmeier I. Régnard K. Moze-Henry N. Yadan J. C. Chaudiere J. (1998). Reactions of 1-methyl-2-phenylindole with malondialdehyde and 4-hydroxyalkenals. Analytical applications to a colorimetric assay of lipid peroxidation. Chem. Res. Toxicol. 11, 1176–1183. 10.1021/tx9701790 9778314

[B15] Ghaly G. Tallima H. Dabbish E. Badr ElDin N. Abd El-Rahman M. K. Ibrahim M. A. (2023). Anti-cancer peptides: Status and future prospects. Molecules 28, 1148. 10.3390/molecules28031148 36770815 PMC9920184

[B16] Horii Y. Shen J. Fujisaki Y. Yoshida K. Nagai K. (2012). Effects of l-carnosine on splenic sympathetic nerve activity and tumor proliferation. Neurosci. Lett. 510, 1–5. 10.1016/j.neulet.2011.12.058 22240100

[B17] Huang C. Jin H. Qian Y. Qi S. Luo H. Luo Q. (2013). Hybrid melittin cytolytic peptide-driven ultrasmall lipid nanoparticles block melanoma growth *in vivo* . ACS Nano 7, 5791–5800. 10.1021/nn400683s 23790040

[B18] Hussein M. Arisha A. Mahmoud E. Abdo S. (2021). Mir-140 and Mir-34a as molecular markers for apoptotic brain in sunset yellow and carmoisine intoxicated mice. Zagazig Vet. J. 49, 37–49.‏ 10.21608/zvjz.2021.66481.1141

[B19] Hussein M. M. A. Gaafar S. F. (2022). Histidine-dipeptides in relation to diabetes and obesity. Int. J. Vet. Sci. 11, 221–228. 10.47278/journal.ijvs/2021.093

[B20] Kaloni D. Diepstraten S. T. Strasser A. Kelly G. L. (2022). BCL-2 protein family: Attractive targets for cancer therapy. Apoptosis 28, 20–38. 10.1007/s10495-022-01780-7 36342579 PMC9950219

[B21] Komarov I. V. Tolstanova G. Kuznietsova H. Dziubenko N. Yanchuk P. I. Shtanova L. Y. (2022). Towards *in vivo* photomediated delivery of anticancer peptides: Insights from pharmacokinetic and-dynamic data. J. Photochem Photobiol. B, Biol. 233, 112479. 10.1016/j.jphotobiol.2022.112479 35660309

[B22] Kumar M. Jaiswal R. K. Yadava P. K. Singh R. P. (2020). An assessment of poly (ADP‐ribose) polymerase‐1 role in normal and cancer cells. BioFactors 46, 894–905. 10.1002/biof.1688 33098603

[B23] Li J. Fu H. Xu C. Tie Y. Xing R. Zhu J. (2010). miR-183 inhibits TGF-beta1-induced apoptosis by downregulation of PDCD4 expression in human hepatocellular carcinoma cells. BMC cancer 10, 354–360. 10.1186/1471-2407-10-354 20602797 PMC2909210

[B24] Liu L. H. Zhang X. Z. (2022). Carrier-free nanomedicines for cancer treatment. Prog. Mater Sci. 125, 100919. 10.1016/j.pmatsci.2021.100919

[B25] Livak K. J. Schmittgen T. D. (2001). Analysis of relative gene expression data using real-time quantitative PCR and the 2(-Delta Delta C(T)) Method. Methods 25, 402–408. 10.1006/meth.2001.1262 11846609

[B26] Mao J. Liu S. Ai M. Wang Z. Wang D. Li X. (2017). A novel melittin nano-liposome exerted excellent anti-hepatocellular carcinoma efficacy with better biological safety. J. Hematol. Oncol. 10, 71–74. 10.1186/s13045-017-0442-y 28320480 PMC5359812

[B27] Mei L. Sang W. Cui K. Zhang Y. Chen F. Li X. (2019). Norcantharidin inhibits proliferation and promotes apoptosis via c‐Met/Akt/mTOR pathway in human osteosarcoma cells. Cancer Sci. 10, 582–595. 10.1111/cas.13900 PMC6361574 30520540

[B28] Moskwa P. Buffa F. M. Pan Y. Panchakshari R. Gottipati P. Muschel R. J. (2011). miR-182-mediated downregulation of BRCA1 impacts DNA repair and sensitivity to PARP inhibitors. Mol. Cell. 41, 210–220. 10.1016/j.molcel.2010.12.005 21195000 PMC3249932

[B29] Moulahoum H. Sanli S. Timur S. Zihnioglu F. (2019). Potential effect of carnosine encapsulated niosomes in bovine serum albumin modifications. Int. J. Biol. Macromol. 137, 583–591. 10.1016/j.ijbiomac.2019.07.003 31276721

[B30] Naderinezhad S. Amoabediny G. Haghiralsadat F. (2017). Co-delivery of hydrophilic and hydrophobic anticancer drugs using biocompatible pH-sensitive lipid-based nano-carriers for multidrug-resistant cancers. RSC Adv. 7, 30008–30019. 10.1039/C7RA01736G

[B31] Nasseri B. (2005). Effect of cholesterol and temperature on the elastic properties of niosomal membranes. Int. J. Pharm. 300, 95–101. 10.1016/j.ijpharm.2005.05.009 16006080

[B32] Prakash M. D. Fraser S. Boer J. C. Plebanski M. de Courten B. Apostolopoulos V. (2021). Anti-cancer effects of carnosine—A dipeptide molecule. Molecules 26, 1644. 10.3390/molecules26061644 33809496 PMC8002160

[B33] Rani M. Kumari R. Singh S. P. Devi A. Bansal P. Siddiqi A. (2023). MicroRNAs as master regulators of FOXO transcription factors in cancer management. Life Sci. 321, 121535. 10.1016/j.lfs.2023.121535 36906255

[B34] Ren L. H. Chen W. X. Li S. He X. Y. Zhang Z. M. Li M. (2015). MicroRNA-183 promotes proliferation and invasion in oesophageal squamous cell carcinoma by targeting programmed cell death 4. BJC 111, 2003–2013. 10.1038/bjc.2014.485 PMC4229630 25211657

[B35] Rinaldi F. Del Favero E. Rondelli V. Pieretti S. Bogni A. Ponti J. (2017). pH-sensitive niosomes: Effects on cytotoxicity and on inflammation and pain in murine models. J. Enzyme Inhib. Med. Chem. 32, 538–546. 10.1080/14756366.2016.1268607 28114822 PMC6010110

[B36] Shehata Y. Rafaat N. Gaafar S. F. Gaafar S. (2018). Selective Cytotoxic and Chemoprotective effect of nanocurcumin against human HCT-116 cell line. BLJ 14, 85–93. 10.21608/blj.2018.47586

[B37] Tehrani M. H. Bamoniri A. Gholibeikian M. (2018). The toxicity study of synthesized inverse carnosine peptide analogues on HepG2 and HT-29 cells. IJBMS 21, 39–46. 10.22038/IJBMS.2017.23153.5852 29372035 PMC5776435

[B38] Tian J. L. Ke X. Chen Z. Wang C. J. Zhang Y. Zhong T. C. (2011). Melittin liposomes surface modified with poloxamer 188: *In vitro* characterization and *in vivo* evaluation. Pharmazie 66, 362–367. 10.1691/ph.2011.0327 21699070

[B39] Tiwari R. Tiwari G. Lahiri A. Ramachandran V. Rai A. (2022). Melittin: A natural peptide with expanded therapeutic applications. J. Nat. Prod. 12, 13–29. 10.2174/2210315510999201210143035

[B40] Turner M. D. Sale C. Garner A. C. Hipkiss A. R. (2021). Anti-cancer actions of carnosine and the restoration of normal cellular homeostasis. Biochim. Biophys. Acta Mol. Cell. Res. 1868, 119117. 10.1016/j.bbamcr.2021.119117 34384791

[B41] Tutt A. N. Garber J. E. Kaufman B. Viale G. Fumagalli D. Rastogi P. (2021). Adjuvant olaparib for patients with BRCA1-or BRCA2-mutated breast cancer. NEJM 384, 2394–2405. 10.1056/NEJMoa2105215 34081848 PMC9126186

[B42] Wang A. Zheng Y. Zhu W. Yang L. Yang Y. Peng J. (2022). Melittin-based nano-delivery systems for cancer therapy. Biomolecules 12, 118. 10.3390/biom12010118 35053266 PMC8773652

[B43] Wolf P. Schoeniger A. Edlich F. (2022). Pro-apoptotic complexes of BAX and BAK on the outer mitochondrial membrane. Biochim. Biophys. Acta - Mol. Cell. Res. 1869, 119317. 10.1016/j.bbamcr.2022.119317 35752202

[B44] Yaacoub C. Rifi M. El-Obeid D. Mawlawi H. Sabatier J. M. Coutard B. (2021). The cytotoxic effect of *Apis mellifera* venom with a synergistic potential of its two main components—melittin and PLA2—on colon cancer HCT116 cell lines. Molecules 26, 2264. 10.3390/molecules26082264 33919706 PMC8070685

[B45] Yadavar-Nikravesh M. S. Ahmadi S. Milani A. Akbarzadeh I. Khoobi M. Vahabpour R. (2021). Construction and characterization of a novel Tenofovir-loaded PEGylated niosome conjugated with TAT peptide for evaluation of its cytotoxicity and anti-HIV effects. Adv. Powder Technol. 32, 3161–3173. 10.1016/j.apt.2021.05.047

[B46] Ye R. Zheng Y. Chen Y. Wei X. Shi S. Chen Y. (2021). Stable loading and delivery of melittin with lipid-coated polymeric nanoparticles for effective tumor therapy with negligible systemic toxicity. ACS Appl. Mater Interfaces 13, 55902–55912. 10.1021/acsami.1c17618 34793125

[B47] Zhang L. Quan H. Wang S. Li X. Che X. (2015). MiR-183 promotes growth of non-small cell lung cancer cells through FoxO1 inhibition. Tumor Biol. 36, 8121–8126. 10.1007/s13277-015-3550-8 25983004

[B48] Zoppo L. Del Zoppo L. Morelli G. Condorelli D. F. Barresi V. Musso N. (2016). Liposome antibody–ionophore conjugate antiproliferative activity increases by cellular metallostasis alteration. MedChemComm 7, 2364–2367. 10.1039/C6MD00461J

